# Cross-cohort convergence of a microglial transcriptional state balance in idiopathic autism and Dup15q syndrome

**DOI:** 10.3389/fpsyt.2026.1952880

**Published:** 2026-09-23

**Authors:** Aojie Lian, Yingmei Yang, Yizheng Jiang, Yiping Liu

**Affiliations:** 1 National Health Commission Key Laboratory of Birth Defect for Research and Prevention, Hunan Provincial Maternal and Child Health Care Hospital, Changsha, Hunan, China; 2 Hunan Provincial University Key Laboratory of the Fundamental and Clinical Research on Neurodegenerative Diseases, Changsha Medical University, Changsha, Hunan, China; 3 The Third Xiangya Hospital of Central South University, Changsha, Hunan, China; 4 School of Life Sciences, Central South University, Changsha, Hunan, China

**Keywords:** autism spectrum disorder, cross-cohort convergence, Dup15q syndrome, microglia, post-mortem cortex, single-nucleus RNA sequencing, transcriptional state

## Abstract

**Background:**

Autism spectrum disorder (ASD) encompasses diverse etiologies, creating a need for molecular measurements that retain biological meaning across cortical resources. We examined a predefined transcriptional balance within microglia-annotated nuclei and asked which expression component accounted for its disease-associated direction.

**Methods:**

Population A and Population B were defined as PsychENCODE/Wamsley clusters 0 and 2. A primary all-donor signature comprised 150 genes per direction derived from 44 donors with at least 10 nuclei in each population; a control-derived signature used 20 eligible controls. The same gene lists were projected separately into Velmeshev idiopathic ASD and Perez Dup15q data. Associations were evaluated using donor-level regression or donor-random-intercept models, with component and sensitivity analyses.

**Results:**

Primary B-minus-A coefficients were positive in PsychENCODE (beta=0.058, P = 0.247, Benjamini–Hochberg q=0.247), Velmeshev (beta=0.154, P = 0.104, q=0.156), and Perez Dup15q (beta=0.151, P = 0.014, q=0.041). Dup15q provided the clearest primary statistical evidence; the idiopathic ASD estimates were directionally concordant but individually nonsignificant. External Population A coefficients were negative (Velmeshev beta=−0.157; Perez beta=−0.147), whereas Population B estimates were closer to zero. Positive B-minus-A direction was retained across alternative signature derivation, Perez cell definitions, tested technical adjustments, contamination/doublet exclusion masks, conservative gene exclusion, and all donor-removal refits within each external resource.

**Conclusion:**

A predefined state-balance measurement identifies a shared direction across the analyzed ASD-related resources, with the clearest evidence in Dup15q. Component analysis favors lower Population A-associated expression over a generalized increase in Population B-associated expression. This defines an interpretable molecular phenotype for mechanistic follow-up without assigning either population to a purified cell subtype.

## 1 Introduction

Autism spectrum disorder (ASD) is not a single molecular disease. Rare disruptive coding variants, recurrent copy-number variants (CNVs), and common polygenic variation all contribute to ASD risk (1–3), and functional genomic studies indicate convergence on neurodevelopmental, transcriptional, chromatin-regulatory, and synaptic systems (4). The practical consequence is that a useful molecular phenotype in ASD should not depend on one etiologic route or on one cohort-specific gene list. The more consequential question is whether heterogeneous causes converge on a reproducible biological direction that can be measured in distinct human brain datasets.

Post-mortem cortical studies have repeatedly shown that such convergence is plausible. Bulk transcriptomic analyses have identified coordinated changes in neuronal, synaptic, splicing, and glial-immune programs across ASD cortex (5–9), while single-cell and single-nucleus ASD studies have resolved those changes into cell-type-specific contexts (10–12). These studies transformed the field from asking whether the ASD cortex is molecularly altered to asking which cellular programs carry the alteration, how consistently they recur across donors and regions, and whether they represent categorical cell types or continuous state changes.

Microglia are an especially informative test case because their normal biology is intrinsically state-dependent. During development, microglia participate in synaptic pruning, complement-dependent elimination, activity-dependent circuit remodeling, and learning-related synapse formation (13–17). Human ASD studies have also reported altered microglial density, neuroglial activation, and immune-related cortical transcriptional signals (18–20). Yet these observations do not imply a universal ‘activated microglia’ subtype. Microglial transcription varies with developmental stage, local tissue environment, inflammatory cues, and disease context (21–25). A dimensional state axis is therefore better suited to human post-mortem data than a claim that every ASD brain contains one discrete disease-specific microglial population.

This distinction matters because relative scores can encode biologically different processes. If a score is defined as Population B minus Population A, a higher value can arise because Population B increases, Population A decreases, or both. Those alternatives imply different downstream hypotheses. A generalized increase in Population B would suggest induction or expansion of a B-associated program. A decrease in Population A would instead suggest attenuation of a molecular context that normally occupies the opposite side of the axis. Decomposing the contrast is therefore not a statistical afterthought; it is the step that converts a cross-cohort score into a biologically interpretable result.

A second challenge is transportability. Gene-level disease effects are sensitive to sequencing depth, normalization, cell abundance, region, and feature detection. A predefined score derived in one resource can be more robust if it summarizes coordinated expression and is then frozen before projection. The critical test is not whether every gene behaves identically in every dataset, but whether the same predefined axis retains direction across cohorts, survives reasonable alternative definitions, and produces coherent component behavior. This logic is especially relevant to ASD, where clinical and etiologic heterogeneity makes exact reproduction of every local effect less informative than convergence on a reproducible molecular dimension (26–28).

An informative cross-resource test should preserve the molecular quantity being compared. Discovering states and disease-associated genes separately in each dataset can establish local biology, but it answers a different question from applying one specified measurement to a new resource. Here, Population A/B definitions and gene lists were fixed before external projection. The external analyses therefore evaluate the direction of the same expression contrast rather than rediscovering a similar-looking cluster after re-optimization. This design provides a practical way to compare heterogeneous cortical resources while retaining their original sampling and processing structures.

A third challenge is the biological unit of inference. Thousands of nuclei from one donor do not constitute thousands of independent disease replicates. Cell-level testing can therefore generate apparently precise results while ignoring the true sampling structure. Donor-aware pseudobulk and mixed-effects approaches provide substantially better control of pseudoreplication in multi-subject single-cell studies (29–32). We consequently built the analysis around donor support from the beginning: signatures were derived from donors represented in both populations, PsychENCODE disease association was tested at donor level, and repeated donor-region observations in external cohorts were modeled with donor random intercepts.

Genetically defined ASD syndromes provide a particularly informative stress test for molecular convergence. Dup15q syndrome is associated with ASD, intellectual disability, epilepsy, and altered neurodevelopment, and recent single-cell work has documented developmental and postnatal molecular changes across cortical cell types (12). A transcriptional axis derived outside the Dup15q dataset can therefore ask a stronger question than within-cohort differential expression alone: does a predefined microglial molecular direction identified in idiopathic ASD-related resources also appear in a genetically coherent ASD syndrome? If so, the result supports cross-etiology convergence without requiring the etiologies to be identical.

Each resource has a distinct role in this comparison. PsychENCODE supplies donor-supported gene rankings and an internal association estimate; Velmeshev evaluates the specified scores in an external idiopathic ASD resource; and Perez evaluates the same scores in Dup15q. We compare the direction and component structure of these resource-specific estimates rather than treating their raw expression scales as interchangeable. This separation between measurement, interpretation, and statistical inference is central to the study design.

The Population A/B axis used here emerged from the current PsychENCODE/Wamsley microglial analysis space. We intentionally retain neutral labels rather than assigning either population to a canonical microglial subtype. In this study, a transcriptional state refers to a reproducible pattern of coordinated gene expression observed within microglia-annotated nuclei; it is treated as a continuous molecular context rather than a purified, discrete, or canonical cell subtype. This wording is important because human single-nucleus data may incompletely capture microglial activation-associated transcripts (33), while cross-lineage and ambient-RNA signals remain additional interpretive concerns in processed post-mortem nuclei.

We designed a derivation-and-projection study with four linked goals: define a donor-supported Population A/B axis; apply fixed primary and control-derived signatures to external idiopathic ASD and Dup15q resources; distinguish lower Population A-associated expression from higher Population B-associated expression; and assess the stability of the fitted direction under specified technical and donor-removal analyses. Together, these steps address whether different ASD contexts share an interpretable direction of transcriptional change, rather than whether they contain an identical categorical microglial subtype.

## 2 Materials and methods

### 2.1 Study design, cohorts, and analysis units

We analyzed three published human post-mortem cortical resources. PsychENCODE/Wamsley provided the derivation dataset and internal ASD-control association analysis, with the resource described by Gandal et al. and Wamsley et al. (9–11). Velmeshev et al. provided the external idiopathic ASD resource (10), and Perez et al. provided the external Dup15q resource (12). No new participants or tissue samples were collected. Population definitions and gene lists were determined in PsychENCODE and were not re-ranked using disease associations in the external resources. The derivation-and-projection framework is shown in **Figure 1A**, and cohort characteristics are summarized in **Supplementary Table 1**.

**Figure 1:**
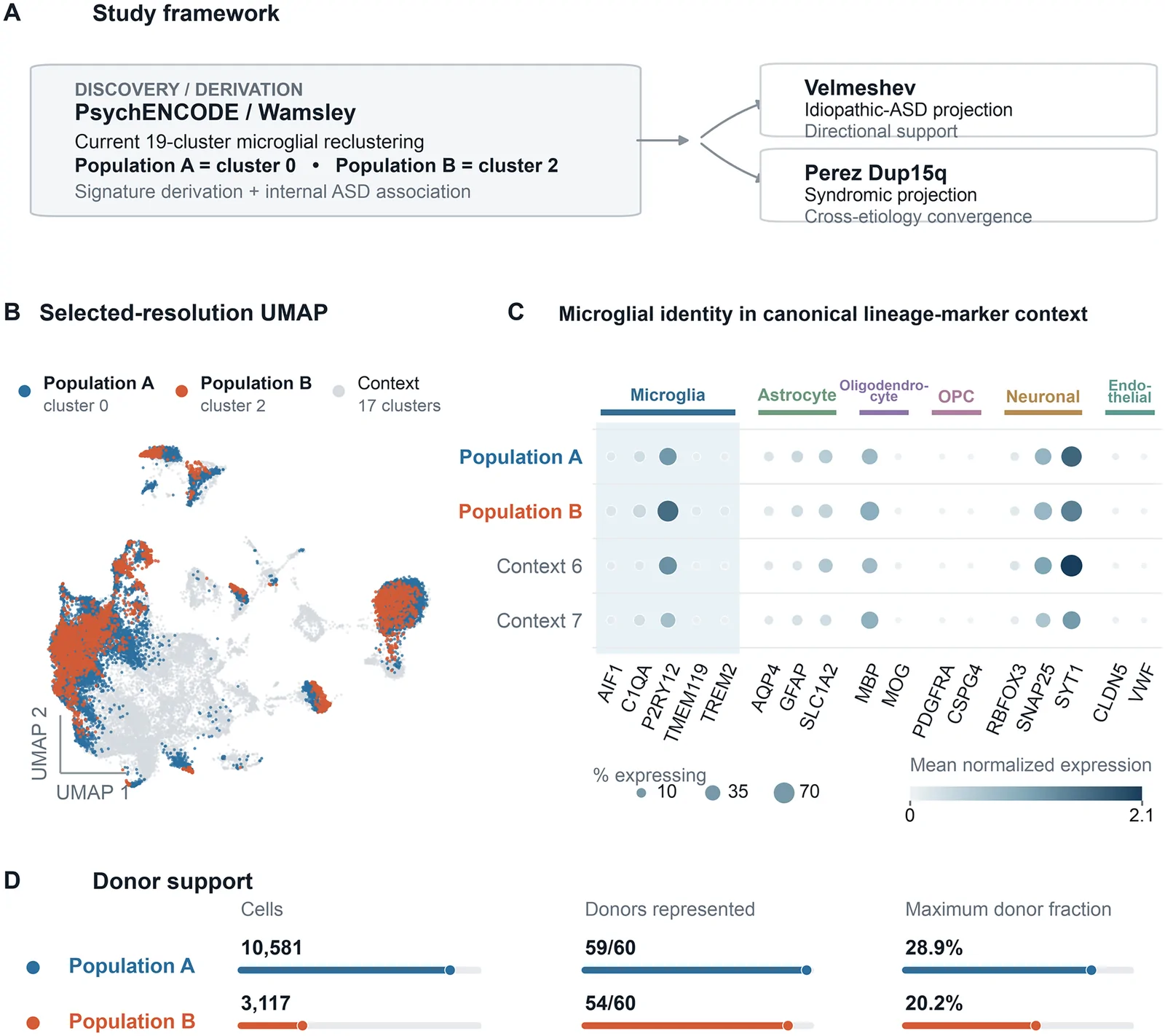
Study framework, lineage-marker context, and donor support for the Population A/B axis. **(A)** PsychENCODE/Wamsley derivation of the Population A (cluster 0) and Population B (cluster 2) signatures, followed by separate projection into Velmeshev idiopathic ASD and Perez Dup15q. The arrows denote analytical projection, not verified between-resource specimen independence. **(B)** Selected-resolution UMAP of the 31,443-nucleus, 19-cluster PsychENCODE analysis space; Population A and Population B are highlighted against the 17 other clusters. **(C)** Representative canonical lineage-marker expression in Population A, Population B, and context clusters 6 and 7. Dot size denotes percentage expressing and color denotes mean normalized expression; values are not z-scored across these clusters. The expanded panel is in **Supplementary Figure 2**. **(D)** Cell totals, donors represented out of 60 cohort donors, and the largest single-donor fraction of each population.

The selected-resolution PsychENCODE object comprised 31,443 post-quality-control (QC) nuclei from 60 donors (31 ASD and 29 controls), distributed across 19 clusters. The A/B analysis pool contained 13,698 nuclei from 59 donors (31 ASD and 28 controls); these 59 donors contributed to the primary donor-level disease model. Forty-four donors met the more restrictive paired-population threshold for signature derivation. The cohort, disease-model, and signature-derivation denominators are therefore 60, 59, and 44, respectively, and refer to different analysis tasks rather than interchangeable sample counts.

Velmeshev contributed 3,331 microglia-annotated cells from 29 donors (15 ASD and 14 controls), represented by 37 sample/region units from anterior cingulate cortex (ACC) and prefrontal cortex (PFC). Perez contributed 27 donors (11 Dup15q and 16 controls), represented by 48 sample/region units from cingulate cortex (CC), frontal cortex (FC), and temporal cortex (TC). PsychENCODE regression used one observation per donor. External models retained sample/region units and included a donor random intercept. Consequently, nuclei and repeated regional samples were not counted as independent disease replicates.

### 2.2 Population definitions, lineage context, and donor support

Population A was defined as selected-resolution cluster 0 and Population B as cluster 2. Population A contained 10,581 nuclei across 59 donors; Population B contained 3,117 nuclei across 54 donors. Their maximum single-donor fractions were 28.9% and 20.2%, respectively (**Figures 1B, D**; **Supplementary Table 2**). These summaries describe representation and concentration of nuclei. They do not, by themselves, establish that an expression contrast is unaffected by donor influence. Paired-donor eligibility and median cell support across minimum-cell thresholds are shown in **Supplementary Figure 1**.

Population A and Population B denote analytical transcriptional programs within microglia-annotated nuclei, not purified or canonical subtypes. **Figure 1C** displays representative microglial markers (AIF1, C1QA, P2RY12, TMEM119, and TREM2) together with astrocytic, oligodendrocytic, oligodendrocyte precursor cell (OPC), neuronal, and endothelial markers. Dot size is the percentage of nuclei expressing the gene, and dot color is mean normalized expression. The values are not z-scored across the four displayed clusters. **Supplementary Figure 2** expands the marker panel and shows marker-set positivity and parent annotations for Population A, Population B, and context clusters 6 and 7. Parent annotations describe the source analysis space rather than an independent purity measurement.

Nucleus-level QC context includes RNA counts, detected-feature counts, Scrublet scores, and the fraction exceeding a Scrublet score of 0.25 (**Supplementary Figure 3**). The percentages above this descriptive threshold were 1.74% for Population A, 0.80% for Population B, 8.82% for context cluster 6, and 1.95% for context cluster 7. The 0.25 threshold defines this QC summary; it is not substituted for the separate stored exclusion masks used in the filtering sensitivity analyses below. Source-cohort QC and downstream sensitivity exclusions are distinguished throughout.

### 2.3 Directional signatures and paired-donor derivation

A directional list is the ordered set of genes associated with one population relative to the other in the donor-paired comparison. A signature comprises the Population A-associated and Population B-associated directional lists used for scoring. The primary all-donor signature comprises the two fixed top-150 lists derived from eligible ASD and control donors. The control-derived sensitivity signature comprises two top-150 lists derived using eligible controls only. It is an alternative derivation, not a treatment group or a second primary hypothesis. Signature definitions, overlap, and target-resource gene coverage are reported in **Supplementary Table 3**.

Expression was aggregated within donor–population strata. A paired contrast compares Population B and Population A from the same donor; pairs do not match an ASD donor to a different control donor. At least 10 nuclei in each population were required for the primary derivation, retaining 44 donors (24 ASD and 20 controls). The control-derived signature used the same criterion in the 20 eligible control donors. For each derivation, the fitted donor-paired contrast was summarized with limma topTable. Genes with logFC > 0 were ranked as Population B-associated and genes with logFC < 0 as Population A-associated; within each direction, genes were ordered by ascending raw P value, then descending absolute logFC, then ascending gene symbol, and the first 150 genes were retained. FDR was recorded but was not used as a preselection cutoff. The exact ordered lists used for all projections are supplied as **Supplementary Data S1–S4**; neither disease-associated re-ranking nor replacement of missing genes was performed in the target resources.

Minimum-cell thresholds of 1, 3, 5, 10, 20, 50, and 100 retained 54, 52, 50, 44, 39, 19, and 8 paired donors, respectively. At the primary 10-cell threshold, 73.3% of the 60-donor cohort was eligible. Alternative derivations used the five- and 20-cell thresholds, while score-size analyses used the top 25, 50, 100, and 150 genes. These were sensitivity analyses, not a search for the most favorable disease association. The complete eligibility grid is provided in **Supplementary Table 4**.

Primary and control-derived Population A lists shared 114 genes (union 186; Jaccard index 0.613), whereas Population B lists shared 48 (union 252; Jaccard index 0.190). Because the 20 controls are included among the 44 primary derivation donors, the derivations are overlapping sensitivity analyses, not independent replications. Their agreement evaluates dependence on the inclusion of ASD donors in gene ranking.

### 2.4 Expression scores and primary disease-association models

A single-nucleus object refers here to an expression matrix together with nucleus-level annotations and donor/sample metadata. Each resource retained its own expression preprocessing and metadata; the three matrices were not combined into a joint integrated embedding. PsychENCODE and Velmeshev scores used the normalized expression in the processed analysis objects. For Perez, counts were converted to log1p counts per 10,000, as documented in the original analysis, before score aggregation. Thus, raw score units are resource-specific. The PsychENCODE model and score-size sensitivities are displayed in **Supplementary Figure 4**; external feature coverage is shown in **Supplementary Figure 5**.

For a nucleus, the Population A score is the arithmetic mean of the available genes in the fixed A-associated list; the Population B score is defined analogously. The balance is B minus A. Scores were averaged over the relevant A/B analysis-pool nuclei for each PsychENCODE donor and over the selected microglial cells within each external sample/region unit. Available genes were retained with equal weight. Primary coverage was 146/150 A genes and 148/150 B genes in Perez, and 144/150 and 135/150 in Velmeshev. Control-derived coverage was 147/150 and 147/150 in Perez, and 145/150 and 140/150 in Velmeshev. Missing target genes were not imputed or replaced.

The PsychENCODE primary disease model was a donor-level linear regression of the top-150 primary balance on diagnosis, age, sex (the recorded Sex_Chromosome covariate), brain region, post-mortem interval (PMI), and RNA integrity number (RIN). It included 59 donors. Unadjusted, age/sex/region-adjusted, batch-adjusted, alternative derivation, and score-size models were sensitivity analyses (**Supplementary Table 5**). The model estimates the association of diagnosis with the average expression balance, not a change in the proportion of A or B nuclei. Observed scores in **Figure 2C** provide descriptive context rather than a separate inferential test.

**Figure 2:**
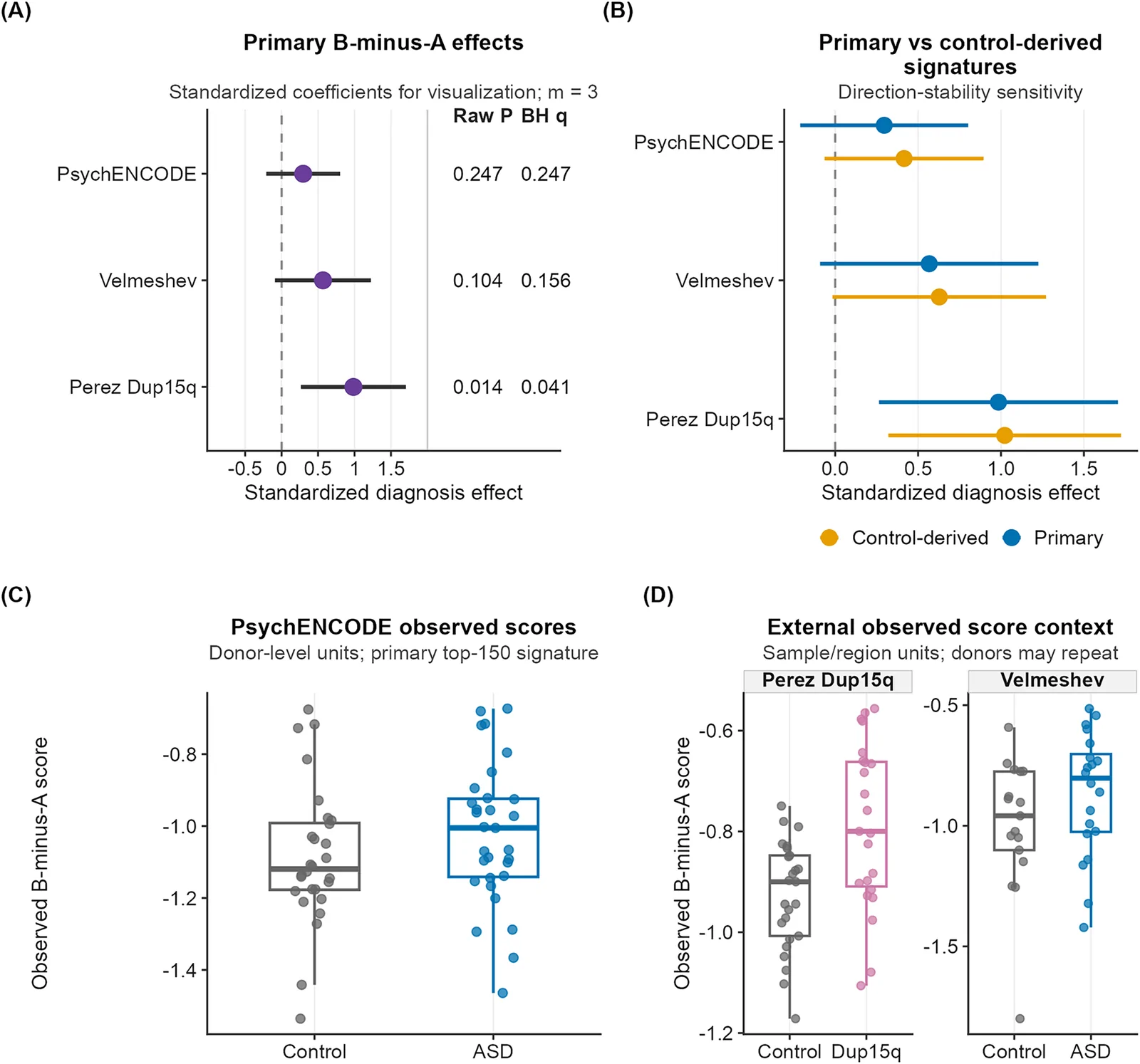
Primary population B-minus-A associations and observed score context. **(A)** Primary standardized diagnosis coefficients and 95% confidence intervals; the adjacent raw P and BH q values come from the three raw-scale primary tests (m=3). **(B)** Primary and control-derived standardized estimates and 95% confidence intervals; these alternative derivations assess direction stability. **(C)** Observed primary top-150 PsychENCODE scores, with one point per donor. **(D)** Observed Perez and Velmeshev scores, with one point per sample/region unit; donors may repeat. Boxplots summarize the observed distributions and do not replace adjusted models. Standardized effects facilitate visualization; primary inference uses raw-scale models.

For external B-minus-A models, the outcome is the sample/region mean B-minus-A score. Velmeshev fixed effects were diagnosis, age, sex, and region; Perez fixed effects additionally included PMI and RIN. Both external models included a donor random intercept. Diagnosis compared ASD with control in PsychENCODE and Velmeshev, and Dup15q with control in Perez. The three primary raw-scale estimates and their primary-family adjusted P values are reported in **Supplementary Table 6**; **Figure 2A**. The control-derived estimates in **Figure 2B** remain sensitivity analyses. Points in **Figure 2D** are sample/region units, so a donor can contribute more than one point.

Population A, Population B, and B-minus-A were fitted as separate outcomes. Component estimates were used to characterize the balance, rather than to infer generalized activation from the sign of a difference score alone (**Supplementary Table 7**). In a mixed model, each outcome can have separately estimated variance components; the fitted B-minus-A coefficient therefore need not equal subtraction of the two separately fitted component coefficients. Outcome-specific standardized coefficients likewise must not be subtracted to reconstruct the standardized balance.

### 2.5 Cell definitions, technical sensitivity, and donor/region analyses

The Perez primary microglial definition (Primary MG) used the released MG cell-type label, retaining 28,538 nuclei. The strict definition (Strict MG) additionally required the released Microglia cluster label, retaining 28,394 nuclei. Both definitions contributed 27 donors and 48 sample/region units. Cell-definition sensitivity is reported in **Supplementary Table 8** and **Supplementary Figure 6**.

Capbatch denotes capture/processing batch and Seqbatch denotes sequencing batch. These technical variables were examined because batch structure can covary with anatomical sampling and diagnosis. In Perez, Capbatch, Seqbatch, and analyzed cell count were added separately to the primary covariate structure, rather than all simultaneously (**Supplementary Table 9**). In Velmeshev, Seqbatch- and cell-count-adjusted models were fitted separately; Capbatch adjustment was not estimable because of collinearity. The estimable technical adjustments therefore differed between resources. Missing covariates were not imputed across resources.

Leave-one-donor-out (LODO) analysis removed all sample/region units belonging to one donor and refitted the corresponding model, yielding 29 refits per signature in Velmeshev and 27 in Perez. Individual refits and summary statistics are reported in **Supplementary Table 10**, with the external sensitivity displays in **Supplementary Figures 6**, **7**. Six control-label strings occur in both external resources (4341, 5163, 5242, 5387, 5893, and 5958). Because matching labels alone do not establish specimen identity, we treated this as a conservative worst-case robustness scenario rather than as evidence of donor overlap. All Perez sample/region units carrying these six labels were removed together and the unchanged base model was refitted, leaving 21 donors (11 Dup15q, 10 controls) and 41 sample/region units. This candidate control-label exclusion is sensitivity-only and does not replace the prespecified full-cohort analysis (**Supplementary Tables 6**, **7**). LODO evaluates influence within each resource, whereas this additional analysis tests whether the Perez result depends on observations carrying these candidate labels.

Regional heterogeneity was assessed by a diagnosis-by-region interaction test, comparing a region-invariant diagnosis association with a model allowing diagnosis effects to vary by region. Region-stratified coefficients were displayed separately for descriptive context. A nominal association in one region and a nonsignificant association in another was not interpreted as an interaction. Formal tests and stratified estimates are reported in **Supplementary Table 11**; the Velmeshev presentation is shown in **Supplementary Figure 7**.

### 2.6 Statistical scale, multiplicity, and contextual summaries

The prespecified primary family comprises three raw-scale B-minus-A tests for the primary all-donor signature: PsychENCODE, Velmeshev, and Perez Dup15q. Benjamini-Hochberg (BH) correction was applied across these three P values (m=3). Control-derived signatures, component outcomes, alternate cell definitions, technical models, and other sensitivities do not add primary hypotheses. Raw P values for those analyses are reported descriptively unless a separate correction is explicitly identified. For the candidate control-label exclusion sensitivity, a separate sensitivity-only m=3 BH calculation substituted the exclusion Perez P value while retaining the frozen PsychENCODE and Velmeshev P values; it is reported only as robustness context and does not replace the prespecified primary q values (**Supplementary Table 6**). This separation preserves the primary inferential question while allowing secondary results to explain its component structure.

Standardized coefficients in the main effect plots are visualization quantities, not replacement tests. The PsychENCODE coefficient, standard error, and confidence limits were divided by the standard deviation of the corresponding fixed top-150 donor-level outcome. External figures use the corresponding standardized-outcome model rows from the external analysis alongside their matched raw-scale rows; no second scaling was applied. Raw-model P values provide the primary inference. The three outcomes use outcome-specific standardization, and the plots do not constitute a formal test that one resource has a larger disease association than another.

An exploratory fixed-effect summary combined standardized PsychENCODE and Velmeshev B-minus-A estimates using inverse-variance weights: the pooled coefficient is the weighted mean and its standard error is the inverse square root of the summed weights. Perez was excluded. Because PsychENCODE supplies both the gene rankings and one association estimate, this summary is nonconfirmatory and outside the three-test primary family (**Supplementary Table 12**). **Supplementary Figure 8** also places the scores across all 19 selected-resolution clusters; this within-object comparison is contextual, not an external test of cell-type specificity.

### 2.7 Cohort-specific processing and reporting

The complete fixed-effect and random-effect structures, together with estimable technical sensitivities, are summarized in **Supplementary Table 13**. The 59-donor PsychENCODE model uses the A/B pool, whereas the stricter 44-donor threshold is used for gene-list derivation. External aggregation retains each released sample/region identifier before donor-aware modeling. Tissue-quality adjustment is included in PsychENCODE and Perez as specified above, not imported into Velmeshev as an unmeasured variable.

The released Perez matrix used in the analysis contained 36,601 features and 345,624 barcodes. Target-gene counts were extracted from the sparse matrix for the specified microglial barcodes and normalized as log1p counts per 10,000; cell-level signature scores were then averaged within the retained sample units. The primary and strict microglial extraction counts are documented in **Supplementary Table 14**. These are the dimensions of the analyzed release and subset, not a claim that every barcode in the original publication entered the disease models.

### 2.8 Representative markers and recorded exclusion analyses

The representative-marker summary is distinct from the donor-derived signature lists. **Supplementary Table 15** contains 100 A-associated and 100 B-associated genes ranked by the corresponding mean-expression difference, with a gene detected in at least 5% of nuclei in its associated population. These cluster-level descriptive ranks characterize expression context; they are not the 150-gene donor-paired score ranks. Consequently, a gene present in the representative-marker table need not be a member of a predefined score list.

Gene exclusion was defined against the canonical lineage-marker panel used in the analysis. None of the four score lists overlapped the panel’s astrocyte-specific, oligodendrocyte, OPC, or endothelial subsets. SYT1 and SNAP25 were present in both A-associated lists. The conservative Filter-A sensitivity removed those two genes, reducing A lists from 150 to 148 genes while leaving B lists unchanged, and repeated score aggregation and disease-model fitting (**Supplementary Table 16**). Shared context genes were not classified as lineage-exclusive solely because they occur in more than one cell type. Absence of overlap with this specified panel is not an exhaustive test of lineage specificity.

The PsychENCODE filtering analysis reused the recorded neuronal-marker, Scrublet, and combined exclusion masks on the same 13,698-nucleus A/B pool. All A/B nuclei were matched to the stored masks. The retained pools were 12,336, 13,110, and 11,861 nuclei, respectively. Corresponding disease models included 58, 59, and 58 donors; signature membership and the primary model covariates were unchanged. The model coefficients, standard errors, confidence limits, and retained counts are supplied in **Supplementary Table 5**. These analyses assess stability under the recorded exclusions; they do not establish an ambient-RNA-free transcriptome or identify all possible doublets.

## 3 Results

### 3.1 A donor-supported transcriptional axis is defined within microglia-annotated nuclei

Population A/B was represented across most donors in the selected-resolution resource. Population A contained 10,581 nuclei from 59 of 60 donors and Population B contained 3,117 nuclei from 54 donors. The largest donor contributions were 28.9% and 20.2%, respectively; 44 donors met the primary 10-cell paired threshold, including 24 ASD and 20 controls (**Figures 1B, D**; **Supplementary Figure 1**). These counts establish broad representation while also making the unequal contribution of individual specimens visible.

The axis lies within a microglia-annotated analysis space and retains heterogeneous lineage-marker expression. **Figure 1C** presents microglial markers alongside representative non-microglial markers. The expanded dotplot in **Supplementary Figure 2** retains additional astrocytic and neuronal features rather than omitting them from the identity assessment. Population B displays stronger expression of several microglial signaling genes, while Population A combines microglial and neural/tissue-context transcripts. Parent annotations and marker detection support the source annotation, but do not assign either program to a purified cell subtype.

Representative A-associated features include CLU, APOE, and CST3, alongside neural/tissue-context genes; B-associated features include P2RY12, CSF1R, MERTK, INPP5D, PTPRC, DOCK2, PLCG2, and SYK (**Supplementary Table 15**). These examples describe the cluster-expression contrast. They are not a replacement for the four fixed donor-derived signature lists and do not imply that every listed gene is a microglia-specific marker.

Primary and control-derived signatures provide two overlapping derivations of the axis. Their A lists shared 114 genes and their B lists shared 48 genes, with high feature coverage retained in the target resources (**Supplementary Figure 5**; **Supplementary Table 3**). Concordance across these derivations therefore tests sensitivity to gene-list composition without requiring the lists to be identical or treating their donors as independent samples.

### 3.2 The predefined balance is directionally concordant across resources, with the clearest primary evidence in Dup15q

Once frozen and projected, the Population B-minus-A axis moved in the same positive direction in every primary cohort-level test (**Figure 2A**). PsychENCODE produced beta=0.05799 (95% CI -0.04138 to 0.15737, P = 0.2468, BH q=0.2468), Velmeshev produced beta=0.15353 (95% CI -0.02465 to 0.33171, P = 0.1037, q=0.1555), and Perez Dup15q produced beta=0.15063 (95% CI 0.04042 to 0.26084, P = 0.0137, q=0.0412). The ordering is informative: both idiopathic ASD resources point in the same direction, while the genetically defined Dup15q cohort provides the clearest primary statistical evidence and survives the prespecified three-test FDR correction.

The control-derived sensitivity preserved that structure (**Figure 2B**). In Velmeshev, the control-derived B-minus-A coefficient was 0.14532 (95% CI -0.00379 to 0.29442, P = 0.0676). In Perez Primary MG, it was 0.16389 (95% CI 0.05141 to 0.27637, P = 0.00919). Under the Strict MG definition, the primary and control-derived coefficients increased to 0.16182 (P = 0.00975) and 0.17722 (P = 0.00566), respectively. These models are sensitivity analyses rather than additional primary discoveries, and the positive direction was retained under these alternative signature and cell-definition choices.

Observed score distributions provide complementary context without replacing the models. PsychENCODE donor-level scores show broad overlap between diagnostic groups, consistent with a modest internal coefficient, whereas external donor-region units show an upward displacement that is strongest in Perez (**Figures 2C, D**). The point of **Figure 2** is therefore not to claim identical effect sizes across heterogeneous cohorts. It is to show that a predefined molecular axis can be measured in each resource, that all primary estimates share direction, and that the strongest signal occurs in an etiologically coherent ASD syndrome.

The Velmeshev estimate provides a directionally concordant external observation, while its confidence interval includes zero. Perez provides the clearest primary association under the prespecified correction. Taken together, these estimates identify a common observed direction without requiring equivalence of etiologies, raw score scales, or significance thresholds across resources.

Only the three primary raw-scale B-minus-A models enter the BH family. Perez Dup15q reaches q=0.041, whereas the PsychENCODE and Velmeshev primary tests are individually nonsignificant. Control-derived signatures and the cell-definition and technical models are used to assess the stability of this pattern, not to create additional primary discoveries. Thus, the evidence supports resource-level directional convergence with its strongest statistical anchor in Dup15q.

### 3.3 Component decomposition reveals that reduced Population A-associated expression drives the relative shift

The most important biological refinement comes from decomposing B-minus-A into its two components (**Figure 3**). In Velmeshev, the primary Population A coefficient was -0.15661 (95% CI -0.34991 to 0.03668, P = 0.1248), while Population B was -0.00383 (P = 0.6971). In Perez Primary MG, Population A was -0.14663 (95% CI -0.25710 to -0.03616, P = 0.0163), while Population B was +0.01097 (P = 0.7345). The control-derived signature produced the same pattern: Population A was lower and Population B remained close to zero. Strict MG models again reproduced the same component structure (**Supplementary Tables S7**, **S8**).

**Figure 3:**
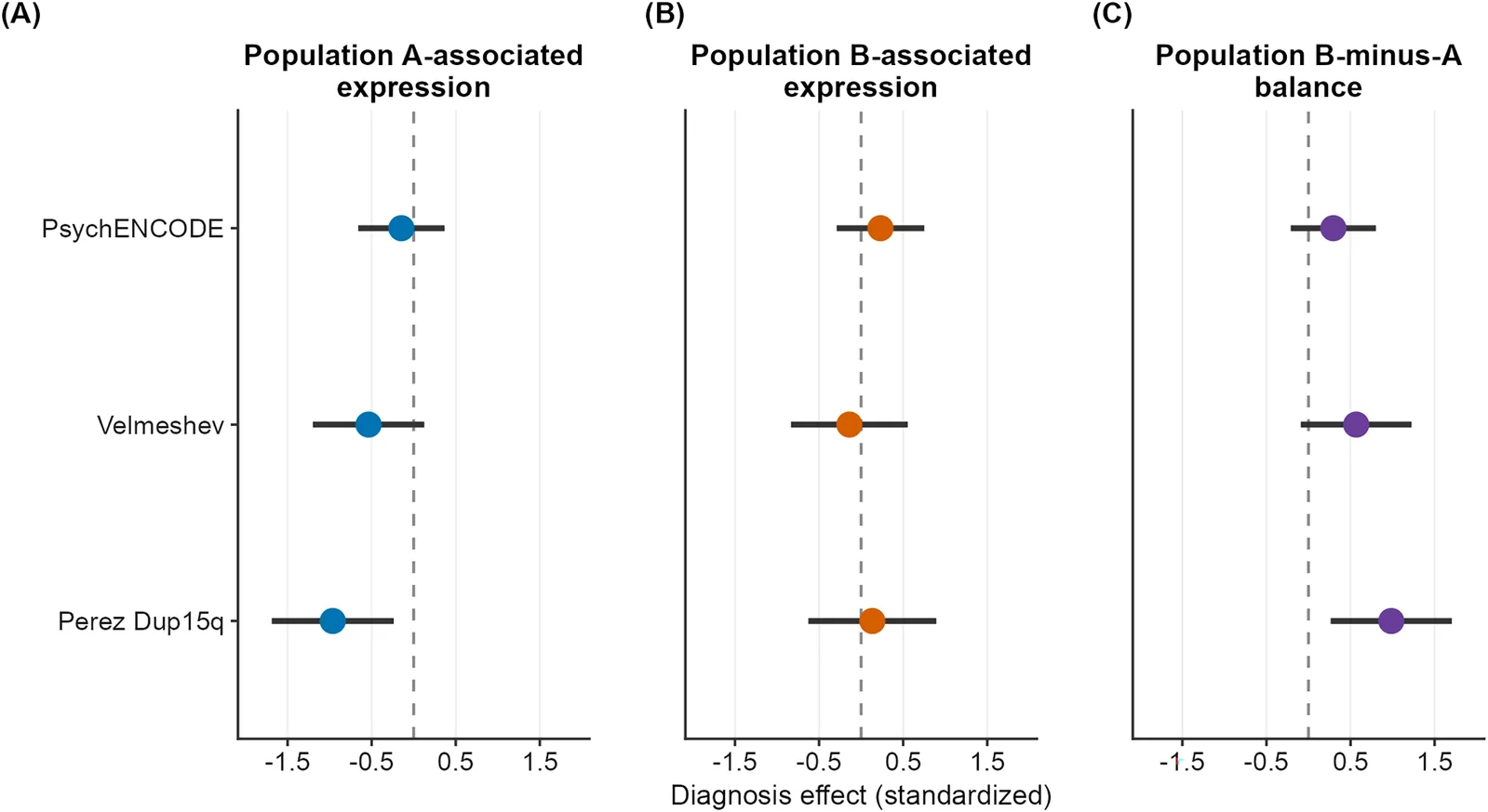
Component analysis of the population A/B balance. Primary standardized diagnosis coefficients and 95% confidence intervals are shown for **(A)** Population A-associated expression, **(B)** Population B-associated expression, and **(C)** Population B-minus-A, in PsychENCODE, Velmeshev, and Perez Dup15q. The external point-estimate pattern favors lower A-associated expression over a generalized B-associated increase. Outcomes are modeled and standardized separately; standardized component estimates should not be subtracted to reconstruct the balance.

The fitted component pattern favors reduced Population A-associated expression over a generalized increase in Population B-associated expression. In both external resources the A coefficient was negative, whereas B estimates were closer to zero. This is an interpretation of separately fitted components, not a causal mediation analysis or a demonstration of equivalence to zero for Population B. The resulting hypothesis concerns attenuation of an A-associated expression context rather than induction of a broad B-associated activation program.

Population A showed greater membership overlap across the all-donor and control-derived lists, sharing 114 of 150 genes compared with 48 of 150 for Population B. The external A estimates were negative under both derivations, and B point estimates remained closer to zero. Agreement in membership and component direction provides complementary support for the A-centered interpretation; neither feature alone establishes a functional mechanism.

This is the principal conceptual advance of the study. The state-balance formulation is useful not only because it transports across datasets, but because it is decomposable. It separates a relative disease axis into biologically different directions and identifies which side actually changes. That distinction narrows the mechanistic question for future experiments from a generic ‘microglial activation’ hypothesis to a more focused question about why a Population A-associated molecular context is attenuated across ASD-related tissues.

### 3.4 The B-minus-A direction is retained under the tested sensitivity analyses

The positive B-minus-A direction was retained under the recorded filtering alternatives (**Figure 4**). In PsychENCODE, neuronal-marker, Scrublet, and combined exclusions changed the primary coefficient from 0.05799 to 0.05201, 0.05249, and 0.04631, respectively (**Figure 4A**). The corresponding retained pools were 13,698, 12,336, 13,110, and 11,861 nuclei at baseline and after each exclusion. The neuronal-marker and combined analyses included 58 rather than 59 donors (**Supplementary Table 5**). The direction was therefore stable under these exclusions, while the estimates remained nonsignificant.

**Figure 4:**
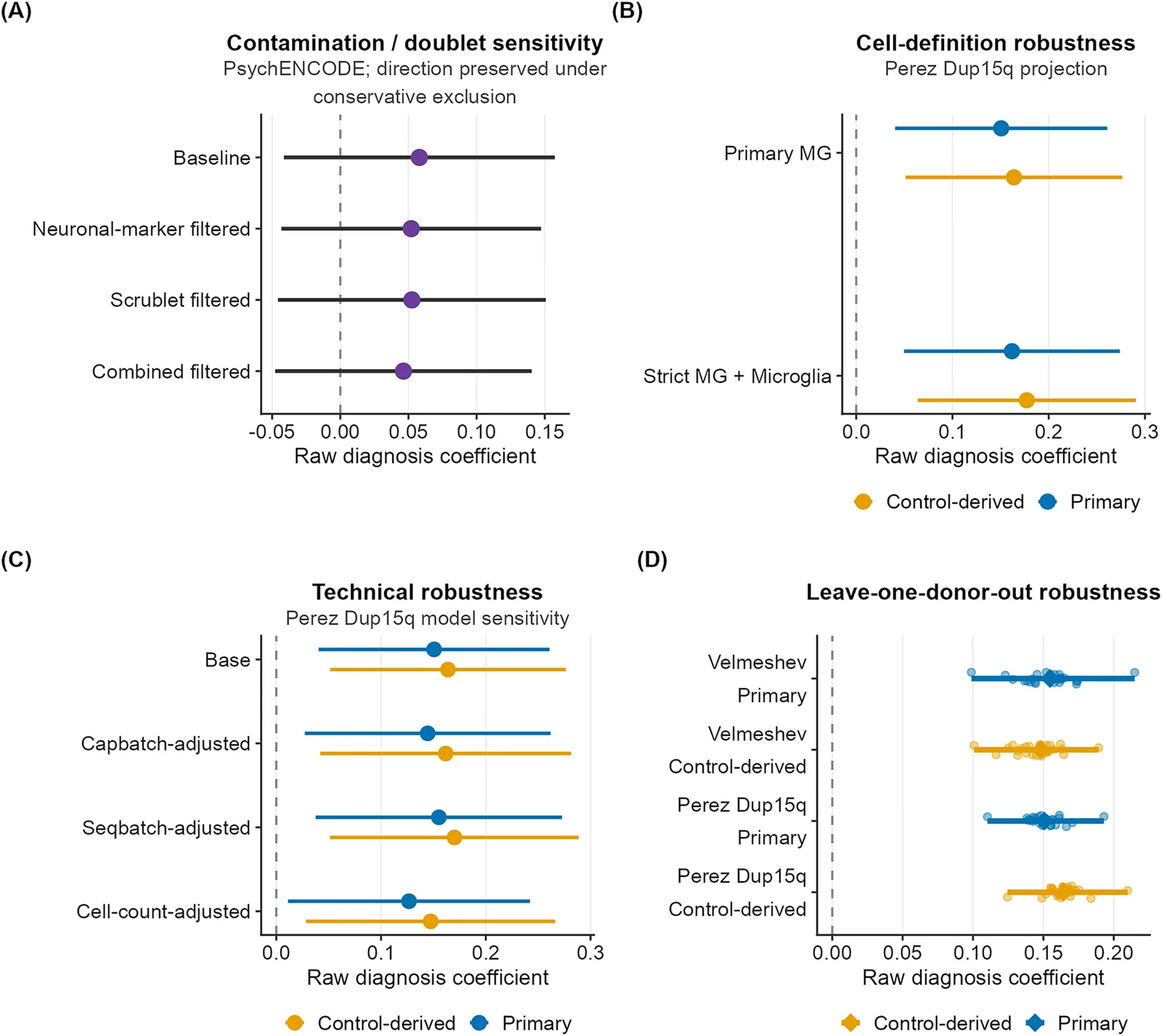
Sensitivity of the population B-minus-A estimates. **(A)** PsychENCODE primary top-150 coefficients and 95% confidence intervals at baseline and after the recorded neuronal-marker, Scrublet, and combined exclusions. **(B)** Perez Primary MG and Strict MG definitions, with primary and control-derived signatures. Strict MG requires both the MG cell-type and Microglia cluster labels. **(C)** Perez base, Capbatch-adjusted, Seqbatch-adjusted, and cell-count-adjusted models. **(D)** Donor-removal coefficient distributions for each external resource and signature. Donor-removal ranges are sensitivity summaries, not confidence intervals for the full-model diagnosis coefficient. Raw-scale coefficients are used in all panels.

The Dup15q result was similarly insensitive to annotation boundary. Primary MG and Strict MG contained 28,538 and 28,394 cells, respectively, yet both definitions produced closely aligned B-minus-A effects for both signatures (**Figure 4B**). Separate technical models also retained the diagnosis direction: for the primary signature, coefficients were 0.15063 in the base model, 0.14452 with Capbatch adjustment, 0.15517 with Seqbatch adjustment, and 0.12670 with cell-count adjustment; the control-derived estimates were 0.16389, 0.16168, 0.17005, and 0.14737, respectively (**Figure 4C**; **Supplementary Figure 6**; **Supplementary Table 9**).

Removing one donor at a time did not reverse the direction of the external diagnosis coefficients. All 29 Velmeshev refits were positive for each signature, as were all 27 Perez refits (**Figure 4D**; **Supplementary Table 10**). This shows that no single within-resource donor deletion reversed the fitted sign. It does not imply that all refits were statistically significant or verify between-resource specimen independence.

Velmeshev primary LODO estimates ranged from approximately 0.099 to 0.215, with a median near 0.155; control-derived estimates ranged from approximately 0.101 to 0.189, with a median near 0.148. Perez primary estimates ranged from approximately 0.110 to 0.193, with a median near 0.151, and control-derived estimates ranged from approximately 0.124 to 0.210, with a median near 0.164. These distributions quantify donor influence without replacing the full-model confidence intervals or the primary tests.

A separate conservative Perez sensitivity removed all observations from the six candidate cross-resource control labels, reducing the dataset to 21 donors (11 Dup15q, 10 controls) and 41 sample/region units. The Primary MG primary-signature B-minus-A coefficient remained positive at beta=0.14575 (95% CI 0.02600 to 0.26549, P = 0.0298), compared with beta=0.15063 in the frozen primary analysis. Population A remained negative (beta=-0.15396, P = 0.0381) and Population B remained near zero (beta=0.00945, P = 0.7920); the control-derived and Strict MG B-minus-A estimates also remained positive, and no fitted comparison reversed direction (**Supplementary Tables 6**, **7**). The separate sensitivity-only multiplicity calculation is reported in **Supplementary Table 6** and does not replace the primary family. Conservative exclusion of SYT1 and SNAP25 from the A-associated lists also retained a positive external B-minus-A direction (**Supplementary Figure 7**; **Supplementary Table 16**). Relative to the specified canonical marker panel, the four fixed lists contained no astrocyte-specific, oligodendrocyte, OPC, or endothelial marker overlap. This gene-list result is separate from the broader expression context in **Supplementary Figure 2**. Formal diagnosis-by-region interactions did not establish regional heterogeneity (**Supplementary Table 11**). The sensitivity results therefore delimit the tested alternatives under which the direction persists; they do not demonstrate cellular purity or exclude unmeasured confounding.

Additional contextual analyses are provided in **Supplementary Figure 8**. Within-object projection across all 19 selected-resolution clusters shows how the predefined scores behave beyond clusters 0 and 2, and an exploratory standardized summary combines the two idiopathic ASD resources without entering the primary inferential family (**Supplementary Table 12**).

## 4 Discussion

### 4.1 A transportable microglial state-balance axis provides a useful level of biological abstraction

The central contribution is an interpretable expression measurement between single-gene differential expression and categorical cell-state annotation. The Population A/B balance is defined from donor-supported programs in one cortical resource, fixed before projection, and measurable in the Velmeshev and Perez resources without reselecting genes in those targets. Its components distinguish lower expression of one program from higher expression of another. This combination is useful for comparing heterogeneous ASD-related tissues without requiring every donor to occupy a new subtype or every resource to reproduce the same differential-expression list.

This level of abstraction is particularly appropriate for microglia. Microglial transcription varies continuously with developmental stage, neuronal activity, lipid and myelin exposure, local inflammatory cues, vascular context, and disease environment (21–25). A rigid subtype label can therefore overstate biological discreteness, while a purely gene-by-gene analysis can be too fragile for cross-cohort projection. The present axis preserves enough structure to be biologically interpretable while remaining flexible enough to move across datasets generated under different sampling and technical conditions.

The balance formulation also creates a useful bridge between descriptive atlases and mechanism. A cluster label is easy to visualize but difficult to compare when clustering resolution changes; a single marker is easy to measure but may be nonspecific; a large differential-expression table is comprehensive but difficult to transport. A two-program balance retains coordinated information while remaining interpretable and portable. It can be projected into a new dataset without recreating the original clustering solution, and it can be decomposed when a disease association appears. Those properties are what allow the present study to move from ‘these cohorts contain different microglial expression patterns’ to the more specific conclusion that they share a direction along one donor-supported axis.

The cross-etiology pattern is also informative. PsychENCODE and Velmeshev, despite different cohorts and analytical contexts, both point toward higher B-minus-A scores. Perez Dup15q shows the same direction with a clearer primary effect that remains significant after the prespecified three-test BH correction. The result is not that all ASD brains share one fixed microglial phenotype. Rather, etiologically distinct ASD contexts can converge along the same molecular direction, with effect magnitude varying across cohorts.

### 4.2 Decomposition converts a descriptive contrast into a biological hypothesis

The component result is the feature that most changes how the cross-cohort signal should be understood. A relative B-minus-A increase sounds, at first glance, like a rise in Population B. The data show otherwise. Population B-associated coefficients remain close to zero in the external cohorts, whereas Population A-associated coefficients are negative and reproducible across the two signature derivations. The state balance therefore changes because one side recedes.

That distinction immediately narrows the mechanistic space. A broad Population B increase would prioritize mechanisms of induction, expansion, or inflammatory activation of the B-associated program. Reduced Population A-associated expression instead raises questions about loss of a tissue-supportive, maintenance, metabolic, or interaction-dependent context represented by Population A. The present data do not assign a single function to Population A, and doing so would be premature because its representative genes span microglial and neural/tissue-context biology. But the direction is sufficiently specific to guide functional work: future experiments should ask what upstream conditions lower the Population A program and what cellular consequences follow.

The stability of Population A rankings across all-donor and control-derived signatures adds another layer. A program that remains similar when disease donors are removed from derivation is less likely to be a simple circular encoding of case-control status. The external disease signal then appears mainly as attenuation of that comparatively stable program. This combination - derivation stability plus external directional reduction - is more informative than attaching a canonical subtype label to either cluster.

**Figure 5** summarizes the proposed model arising from this component pattern. The predefined A- and B-associated programs are projected into the external resources, where the higher relative Population B-minus-A balance is explained primarily by lower Population A-associated expression with comparatively little change in Population B-associated expression. The schematic is explicitly hypothesis-generating and does not imply a direct A-to-B state transition, purified subtype identity, or a proven causal mechanism.

**Figure 5:**
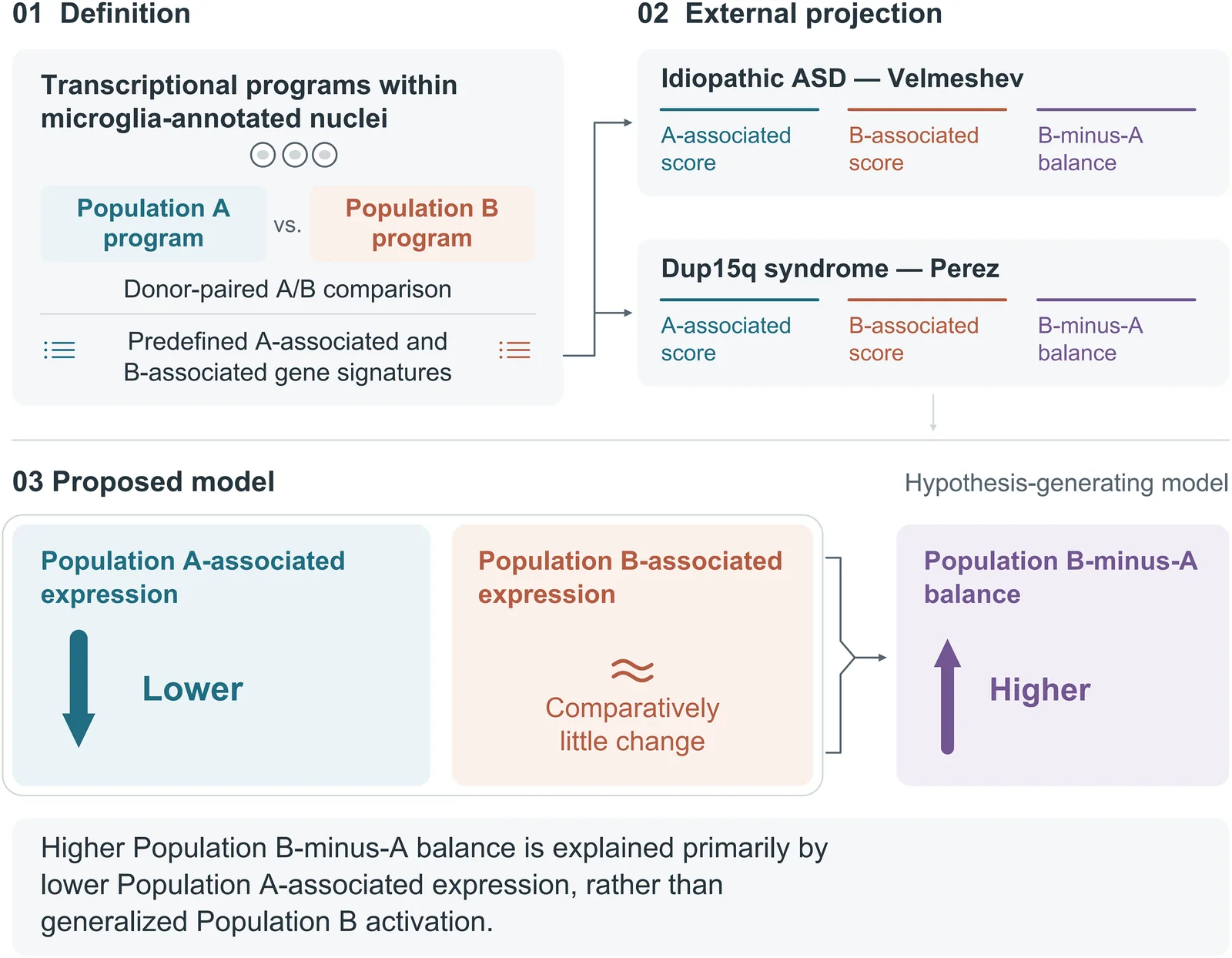
Proposed model for the population A/B transcriptional state-balance shift. Population A and Population B are represented as transcriptional programs measured within microglia-annotated nuclei. Predefined A-associated and B-associated signatures are projected into the Velmeshev idiopathic ASD and Perez Dup15q resources. The external component estimates support a hypothesis-generating model in which Population A-associated expression is lower, Population B-associated expression changes comparatively little, and the resulting Population B-minus-A balance is higher. The schematic summarizes interpretation of the observed component pattern; it does not imply purified subtype identity, direct state transition, or a proven causal mechanism.

### 4.3 Microglial context is supported by convergent lineage and quality-control evidence

Lineage context is particularly important when interpreting a state contrast in post-mortem nuclei. The selected populations originate in a microglia-annotated analysis space, contain microglial marker expression, and retain the source parent annotation. The expanded marker panel also displays astrocytic and neuronal transcripts, including features not present in the reduced main-figure panel. These observations are presented together rather than using the source annotation to override cross-lineage expression. Scrublet summaries and exclusion analyses add sensitivity evidence, but neither assigns a transcript to an intracellular rather than ambient origin.

Population A nevertheless contains neural/tissue-context transcripts among its representative cluster markers, and single-nucleus microglial data can be influenced by ambient RNA or by genuine microglial responses to the surrounding tissue. Accordingly, we describe the axis as transcriptional programs measured within microglia-annotated nuclei. This formulation preserves the microglial context supported by the source data while remaining compatible with the environmental dependence that is intrinsic to microglial identity (24, 25).

The marker-content result is defined relative to the chosen canonical panel, not every gene ever associated with another lineage. It also concerns fixed score lists, whereas **Supplementary Table 15** describes broader cluster-level expression differences. Removing SYT1 and SNAP25 tests whether those two score genes account for the fitted direction; it is not an ambient-RNA correction of the entire expression matrix. The retained direction supports using the A/B balance as an analytical phenotype while keeping its cellular and functional interpretation appropriately specific.

### 4.4 Dup15q functions as an etiologically coherent convergence test

The Perez Dup15q result is not simply another cohort-level association. It tests whether a microglial axis defined outside a recurrent genomic syndrome is displaced in that syndrome in the same direction seen in idiopathic ASD-related resources. The answer is yes, and the effect is the clearest of the three primary tests. This is valuable because syndromic ASD provides a genetically coherent perturbation against the more heterogeneous background of idiopathic ASD.

The clearest primary statistical evidence in Dup15q provides a useful genetically defined setting for follow-up. Its standardized point estimate is larger in the visual comparison, but the study does not formally test between-resource effect-size differences. Differences in diagnosis, age, epilepsy burden, tissue quality, and regional sampling can also affect the estimates. The informative result is that the same specified axis has a positive observed direction in Dup15q and in the idiopathic ASD resources, not that the data establish a quantitative hierarchy of etiologic severity.

Rare variants, CNVs, and common genetic liability can influence overlapping developmental systems through different upstream routes (1, 2, 27). The present expression-based comparison supplies a phenotype with which to investigate that convergence, rather than assigning its observed magnitude directly to a genetic effect. It motivates testing whether specific genetic or environmental perturbations reproduce the negative A-associated component under experimentally controlled conditions.

The distinction between convergence and identical replication remains important for interpretation. Perez is a syndromic cohort with its own developmental, epilepsy, and clinical context, whereas Velmeshev represents idiopathic ASD. The common molecular direction therefore supports a shared axis, while the difference in effect strength is itself compatible with etiologic heterogeneity. The manuscript does not require those contexts to be equivalent in order for the convergence to be informative.

### 4.5 Donor-aware derivation and predefined projection protect against common single-cell failure modes

The design separates gene-list definition from external disease-model fitting and preserves donor structure within each resource. Signatures were derived using donors represented in both populations, PsychENCODE associations were estimated at donor level, and external sample/region models used donor random intercepts. Additional analyses evaluated selected cell definitions, batch variables, cell counts, exclusion masks, donor removal, region, and a conservative worst-case removal of six Perez control labels that also occurred in the Velmeshev resource. In that sensitivity, the Perez control count fell from 16 to 10 while the primary B-minus-A coefficient changed only from 0.15063 to 0.14575, with the Population A-negative/Population B-near-zero component pattern preserved. Thus, the Perez direction is not dependent on observations carrying those labels, regardless of whether the matching identifiers ultimately reflect shared specimens or coincident labeling. Each check has a specific scope; collectively they characterize the settings in which the observed direction is retained.

This matters because multi-subject single-cell studies are particularly vulnerable to pseudoreplication and outcome-guided feature selection (29–32). A pipeline can appear highly significant if cell number rather than donor number drives precision, or if features are repeatedly re-selected in the dataset used for testing. The present framework does the opposite: it fixes the biological axis in one resource, preserves subject-level inference, and asks whether the same quantity behaves coherently elsewhere. That makes the external direction more persuasive than a larger number of within-cohort exploratory comparisons would be.

The control-derived sensitivity reduces dependence of gene ranking on the inclusion of ASD donors. It shares its controls with the primary derivation and therefore is not an independent validation dataset. Its changed gene composition, particularly on the B side, nevertheless provides a useful test of whether the same directional observation requires the original all-donor list. This distinguishes gene-list robustness from specimen independence, which requires separate metadata verification.

### 4.6 Biological implications and experimental priorities

The study delivers a focused molecular phenotype that is immediately suited to mechanistic prioritization because it has a clear direction and a clear component. The next question is no longer ‘are microglia activated in ASD?’ but ‘what lowers the Population A-associated program, and what does that attenuation do to microglial interaction with the developing or diseased cortex?’ Candidate processes include responses to neuronal activity, lipid handling, phagocytic demand, complement and synapse-associated signaling, tissue stress, and region-specific environmental cues - all established dimensions of microglial biology that can be tested without assuming that Population A maps one-to-one onto a canonical state (13–17, 21–23, 34).

Several experimental designs follow directly from the present result. Spatial transcriptomics or multiplexed *in situ* profiling could determine whether low Population A scores localize to particular cortical layers, vascular niches, white-matter interfaces, or sites of altered neuronal pathology. Human microglia-like cells, xenografted human microglia, or neuron-microglia co-culture systems could test whether Dup15q neuronal environments or ASD-relevant perturbations reduce the Population A program. Functional readouts could then ask whether that molecular shift changes phagocytosis, lipid processing, complement responsiveness, cytokine signaling, or synapse-facing behavior.

Because the score is built from cortical nuclei, its immediate value is mechanistic prioritization and cross-dataset comparability. If future studies link the axis to reproducible proteins, metabolites, spatial features, or functional outputs, those downstream measures could then be evaluated in living systems. The present work supplies the directional molecular phenotype needed to make those experiments specific rather than exploratory.

For psychiatric research, this kind of dimensional molecular phenotype may be more useful than a categorical ‘ASD microglia’ label. Clinical ASD diagnoses aggregate individuals with different genetic liabilities, developmental trajectories, epilepsy burden, cognitive profiles, and medication exposures (26–28). A continuous state-balance score can, in principle, be related to those dimensions in future cohorts without requiring the molecular categories to reproduce the diagnostic categories themselves. The immediate next step is to test whether the predefined A/B score tracks clinically or genetically meaningful variation when applied prospectively.

### 4.7 Interpretive scope and validation path

The supported claim is a common observed direction for a specified expression balance across the analyzed resources, with the clearest primary association in Dup15q and a component pattern favoring lower A-associated expression. The idiopathic primary estimates remain nonsignificant; B-component estimates near zero are not evidence of statistical equivalence. Regional subgroup results remain descriptive because formal interactions were nonsignificant, and the pooled idiopathic summary is nonconfirmatory because PsychENCODE contributed the gene rankings. An additional conservative candidate control-label exclusion sensitivity in Perez preserved the B-minus-A direction and the A-negative/B-near-zero component pattern; the prespecified full-cohort analysis remains the primary inference.

These post-mortem resources differ in age, regional sampling, tissue quality, sequencing chemistry, and available clinical covariates. Future validation should apply the specified signatures without re-derivation in an additional, prospectively defined idiopathic ASD cohort with explicit specimen identifiers and matched clinical and technical metadata. Spatial and functional experiments can then test the A-centered hypothesis while separating genuine microglial expression from contributions of the surrounding tissue. This validation path follows directly from the molecular direction identified here.

## 5 Conclusion

A predefined Population A/B balance measured within microglia-annotated nuclei identifies a common observed molecular direction across three ASD-related cortical resources, with the clearest primary evidence in Dup15q. Component analysis favors lower Population A-associated expression rather than generalized Population B activation. The direction is retained under the tested signature, cell-definition, technical, exclusion, and donor-removal analyses. This provides an interpretable molecular phenotype and a focused A-centered hypothesis for experimental investigation of ASD-related cortical state regulation.

## Data Availability

Publicly available datasets were analyzed in this study. The PsychENCODE/Wamsley single-nucleus dataset is available through Synapse (Synapse ID: syn51032009; https://www.synapse.org/#!Synapse:syn51032009/datasets/) and the NIMH Data Archive (Study 3136; DOI: 10.15154/9amm-vr95; https://nda.nih.gov/study.html?id=3136). The Velmeshev autism single-nucleus RNA-sequencing dataset is available through the NCBI Sequence Read Archive under BioProject accession PRJNA434002 (https://www.ncbi.nlm.nih.gov/bioproject/PRJNA434002). The Perez Dup15q dataset is available through the NCBI Sequence Read Archive under BioProject accession PRJNA1017130 (https://www.ncbi.nlm.nih.gov/bioproject/PRJNA1017130), with processed count matrices and metadata available through the UCSC Cell Browser collection “dup15q-cortex-organoids” (https://dup15q-cortex-organoids.cells.ucsc.edu). Derived gene signatures, analysis code, and supporting outputs are available at https://github.com/aojielian/ASD-cortical-microglial-transcriptional-shift-analysis and https://zenodo.org/records/19049731.
