# Supplementary material for: Cross-cohort convergence of a microglial transcriptional state balance in idiopathic autism and Dup15q syndrome
Source: Front Psychiatry. 2026 Sep 23;17:1952880. doi: 10.3389/fpsyt.2026.1952880 (PMC13642789; doi:10.3389/fpsyt.2026.1952880)
Supplement: Supplementary file 1 [file DataSheet1.pdf]

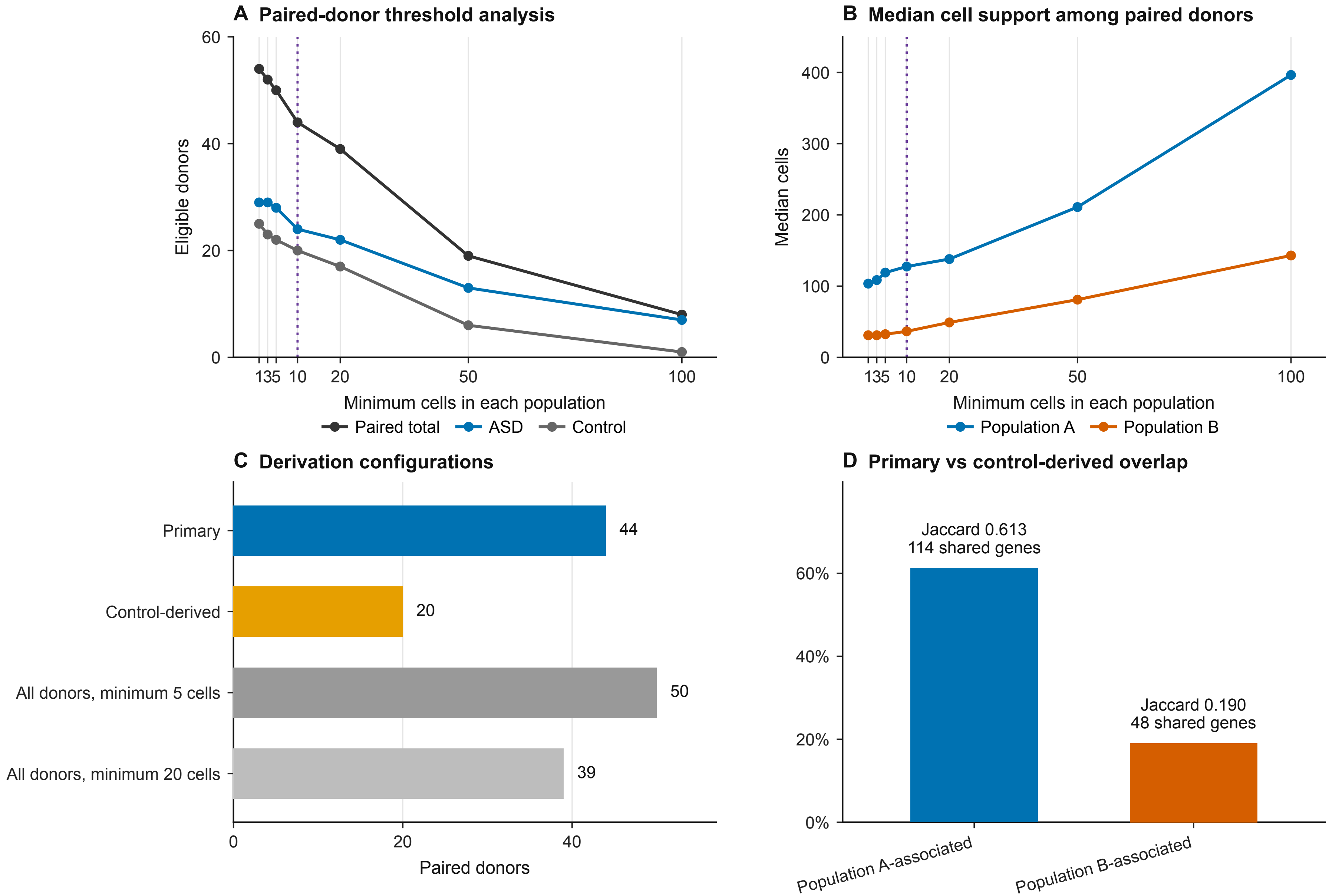

Supplementary Figure S1. Cohort and donor-threshold framework. **(A)** Numbers of eligible paired donors, ASD donors, and Control donors across minimum-cell thresholds applied to both Population A and Population B. **(B)** Median Population A and Population B cell counts among eligible paired donors. The dotted line marks the primary threshold of at least 10 cells in each population. **(C)** Numbers of paired donors contributing to the primary, control-derived, and alternative derivation configurations. **(D)** Primary-versus-control-derived gene-set overlap for Population A-associated and Population B-associated signatures, expressed as Jaccard indices and shared-gene counts. Panels describe the signature-derivation framework; no disease model is fitted in this figure. The primary signature comprises 150 genes per direction derived from 44 paired donors; the 150-gene control-derived signature is a sensitivity analysis.

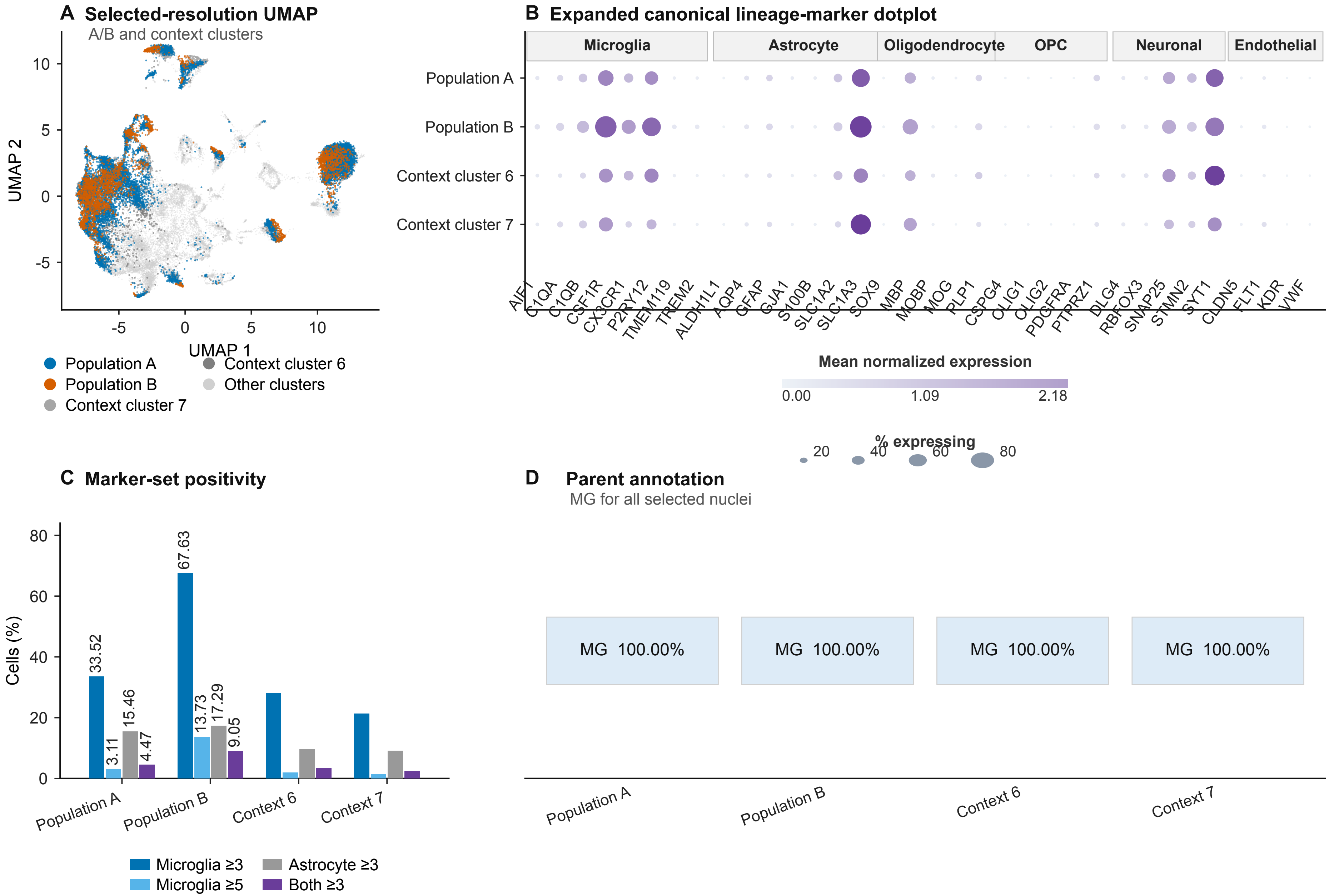

Supplementary Figure S2. Expanded Population A/B identity evidence. **(A)** Selected-resolution UMAP of all PsychENCODE/Wamsley nuclei, highlighting Population A, Population B, and context clusters 6 and 7; each point is one nucleus. **(B)** Expanded canonical lineage-marker dotplot for the same four clusters. Dot color is mean normalized expression and dot size is the percentage of cells expressing the gene; values are not z-scored across clusters. **(C)** Percentages of cells meeting the indicated microglial and astrocytic marker-set positivity thresholds; compact labels on Population A/B bars are rounded only for display from the exact computed values. **(D)** Parent annotation is MG for all nuclei in these selected clusters; this is annotation context, not an independent purity claim. These panels describe transcriptional programs within microglia-annotated nuclei and do not establish a pure or canonical subtype.

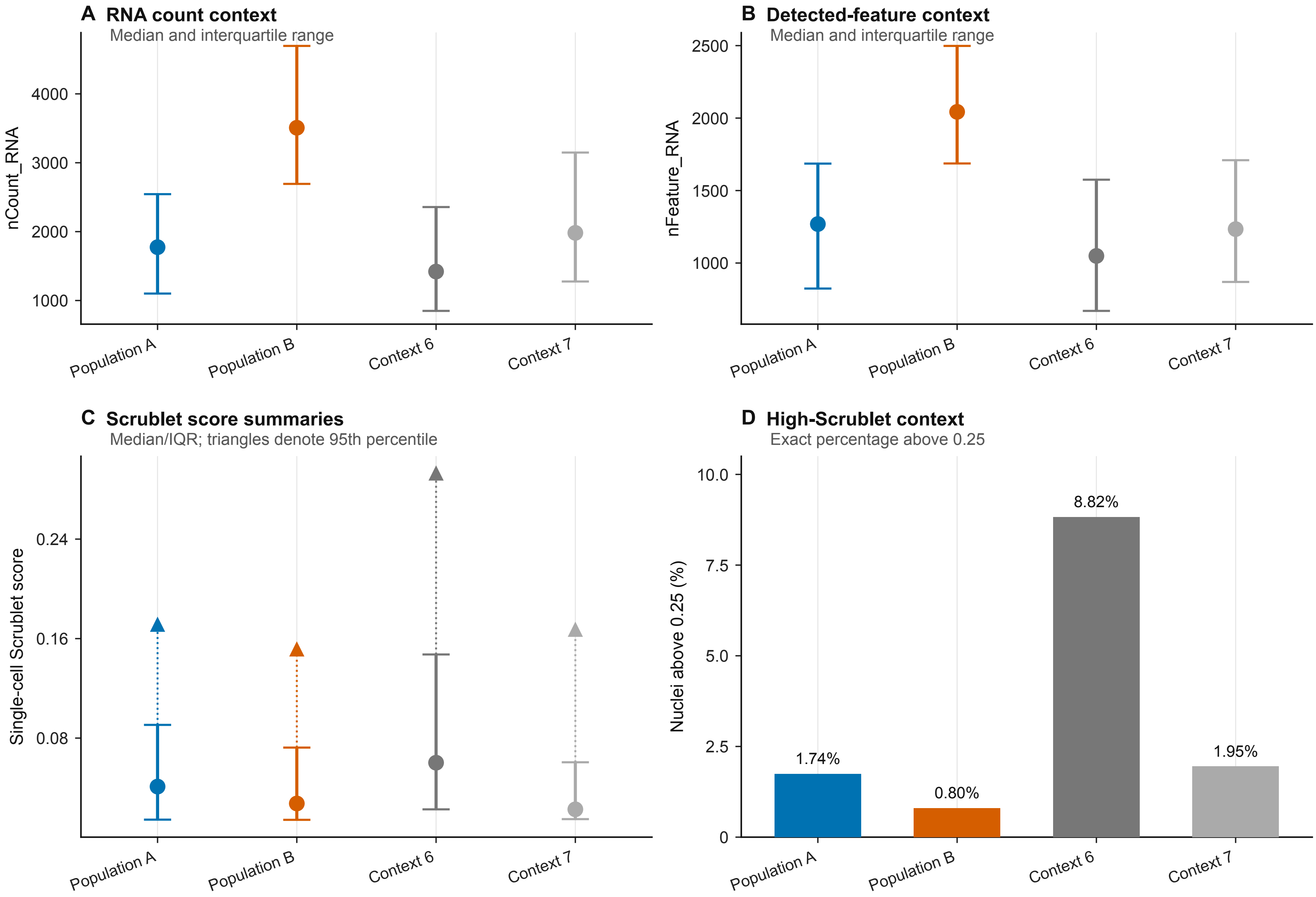

Supplementary Figure S3. QC and conservative contamination context. **(A–C)** Median, interquartile range, and, for Scrublet, 95th percentile of single-cell RNA count, detected-feature count, and Scrublet score in Population A, Population B, and context clusters 6 and 7. **(D)** Exact percentages of nuclei with Scrublet score above 0.25: 1.738966% in Population A, 0.802053% in Population B, 8.823529% in context cluster 6, and 1.948718% in context cluster 7. Each summary is descriptive at the nucleus level. These metrics provide no evidence of Population A-specific doublet enrichment and do not claim that any cluster is free of doublets.

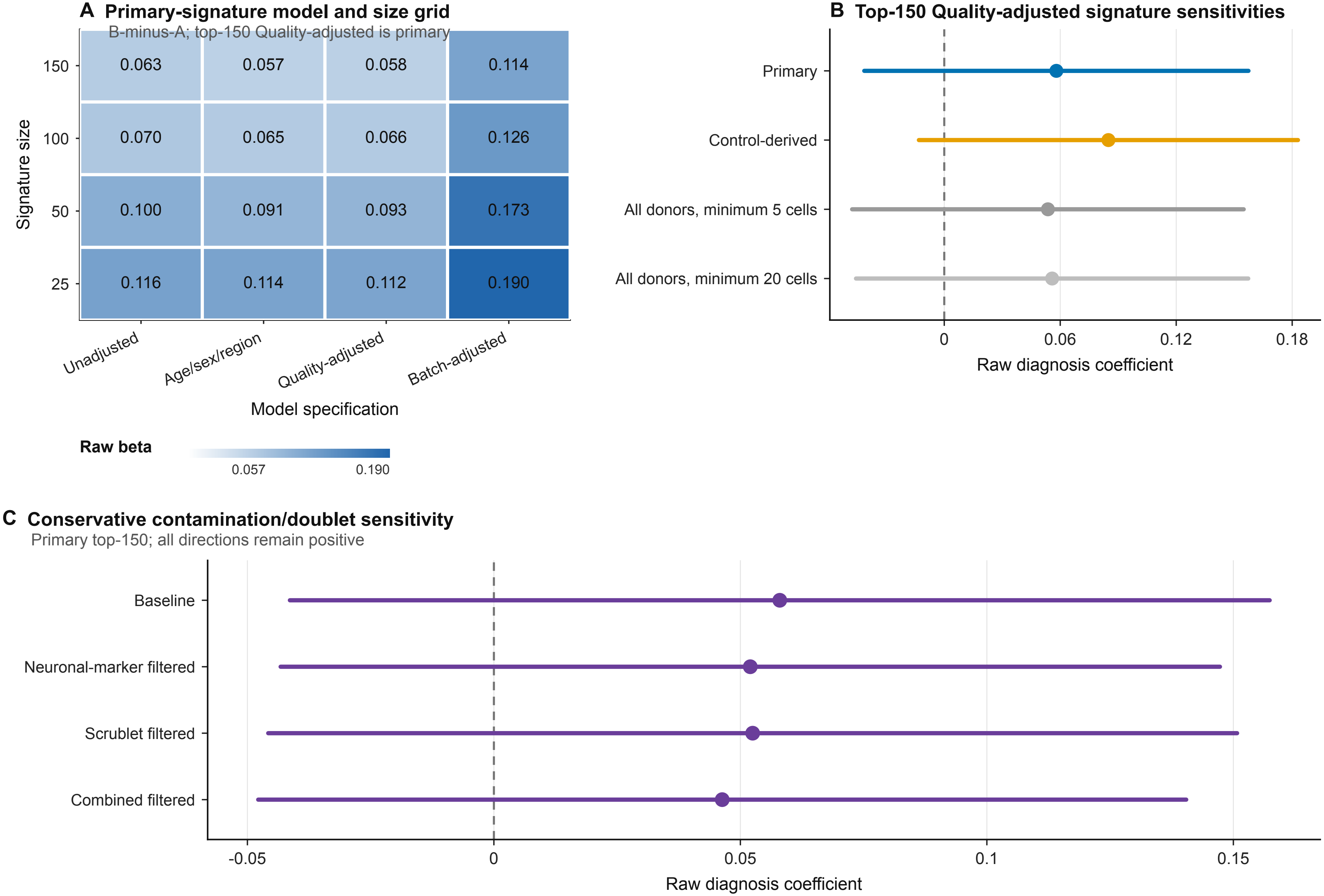

Supplementary Figure S4. PsychENCODE model sensitivity. **(A)** Raw donor-level diagnosis coefficients across primary-signature sizes and PsychENCODE model specifications for the Population B-minus-A score. **(B)** Raw coefficients and 95% confidence intervals from the top-150 quality-adjusted models across derivation configurations. **(C)** Raw coefficients and 95% confidence intervals after baseline, neuronal-marker, Scrublet, and combined filtering. The prespecified primary estimate is the primary all-donor top-150 quality-adjusted linear model, adjusted for diagnosis, age, sex chromosome, brain region, postmortem interval, and RNA integrity number; all other sizes, derivations, and model specifications are sensitivity analyses. Multiple-testing inference is not reassigned to this sensitivity grid; the primary three-test Population B-minus-A BH family remains defined across PsychENCODE, Velmeshev, and Perez Dup15q.

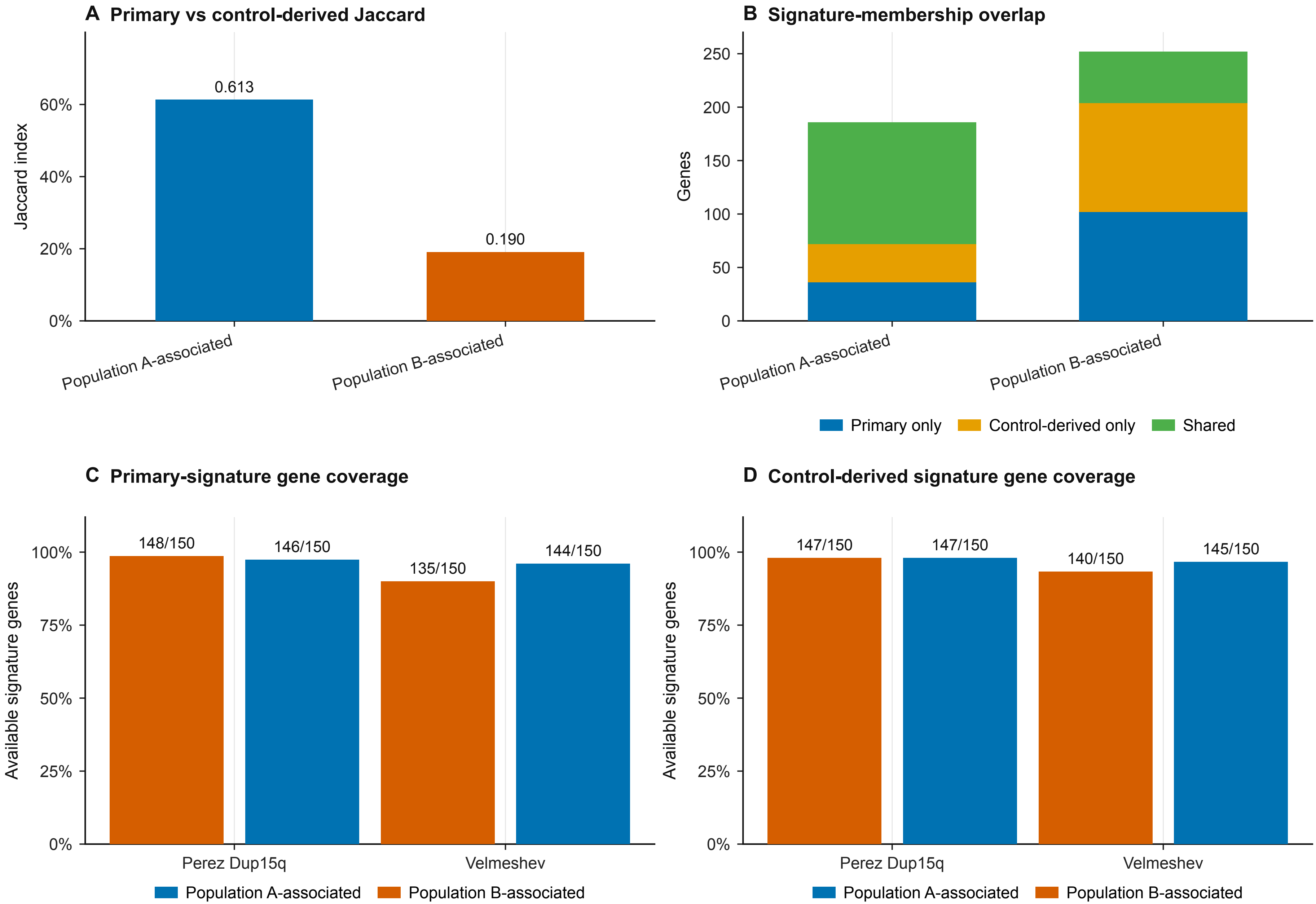

Supplementary Figure S5. Signature stability and cross-dataset gene coverage. **(A–B)** Jaccard similarity and membership overlap between the primary and control-derived 150-gene Population A-associated and Population B-associated signatures. **(C–D)** Numbers and fractions of signature genes available in each projected dataset. Coverage values were verified against the cluster 0/2-derived signatures. The panels are descriptive and contain no disease-effect model or enrichment result.

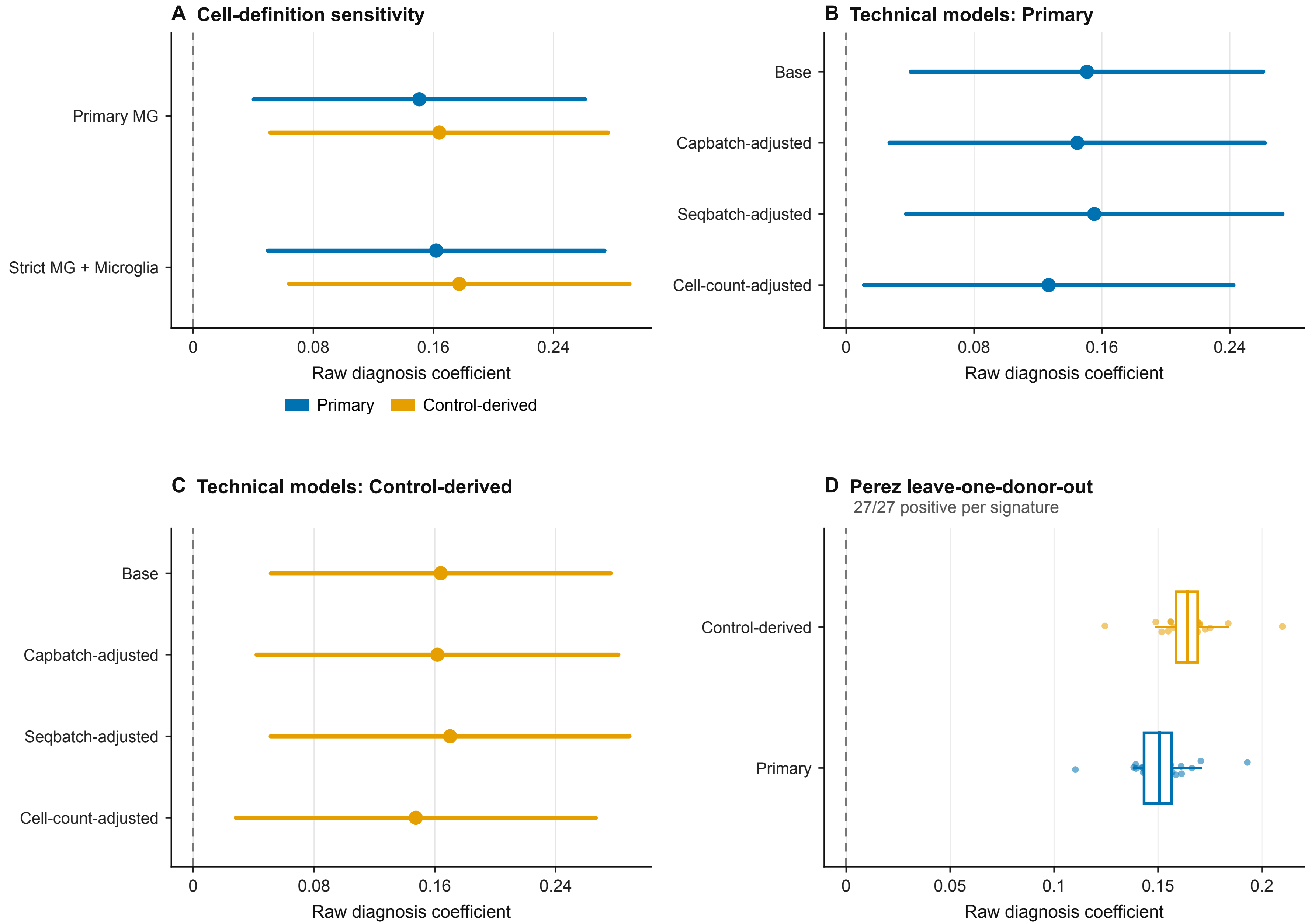

Supplementary Figure S6. Perez Dup15q robustness. All panels use the Population B-minus-A score and raw diagnosis-coefficient scale. **(A)** Primary-MG versus strict-MG cell-definition sensitivity. **(B–C)** Base, Capbatch-adjusted, Seqbatch-adjusted, and cell-count-adjusted mixed models for the primary and control-derived signatures. **(D)** Perez leave-one-donor-out distributions: 27 iterations for each signature, with all 27 beta estimates positive. Points and horizontal intervals in panels A–C are coefficients and 95% confidence intervals from sample/region-level linear mixed models with a donor random intercept; the base model includes diagnosis, age, sex, broad region, postmortem interval, and RNA integrity number. These panels provide Dup15q syndrome-context convergence and technical/donor sensitivity, not a second idiopathic-ASD replication. The primary-versus-control-derived distinction is retained, and inferential multiplicity remains the prespecified three-test primary raw-model family.

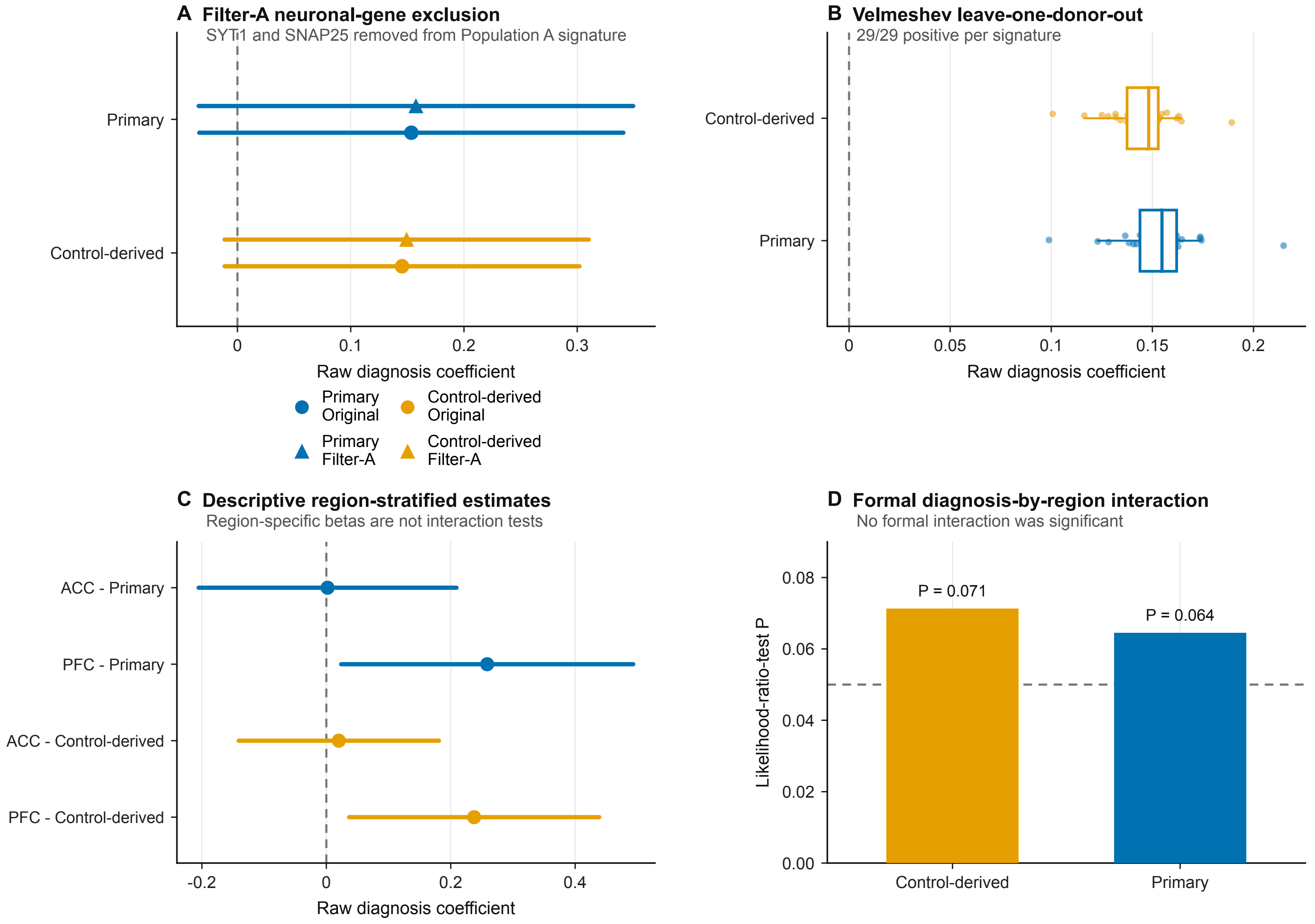

Supplementary Figure S7. Velmeshev robustness and region context. All effect panels use the raw diagnosis-coefficient scale. **(A)** Original and Filter-A estimates after removing SYT1 and SNAP25 from the Population A signature. **(B)** Velmeshev leave-one-donor-out distributions: 29 iterations for each signature, with all 29 beta estimates positive. **(C)** Descriptive ACC- and PFC-stratified coefficients and 95% confidence intervals; these estimates are not interaction tests. **(D)** Formal diagnosis-by-region likelihood-ratio tests for the primary and control-derived signatures. Neither interaction was significant (primary $P = 0.0644$; control-derived $P = 0.0712$). Velmeshev models use sample/region units, with diagnosis, age, sex, and region plus a donor random intercept for the primary mixed model; region-stratified estimates are descriptive linear models. These results are directional idiopathic-ASD support, nonsignificant. The control-derived signature and all robustness analyses are sensitivity analyses, and LODO positivity is not independent replication.

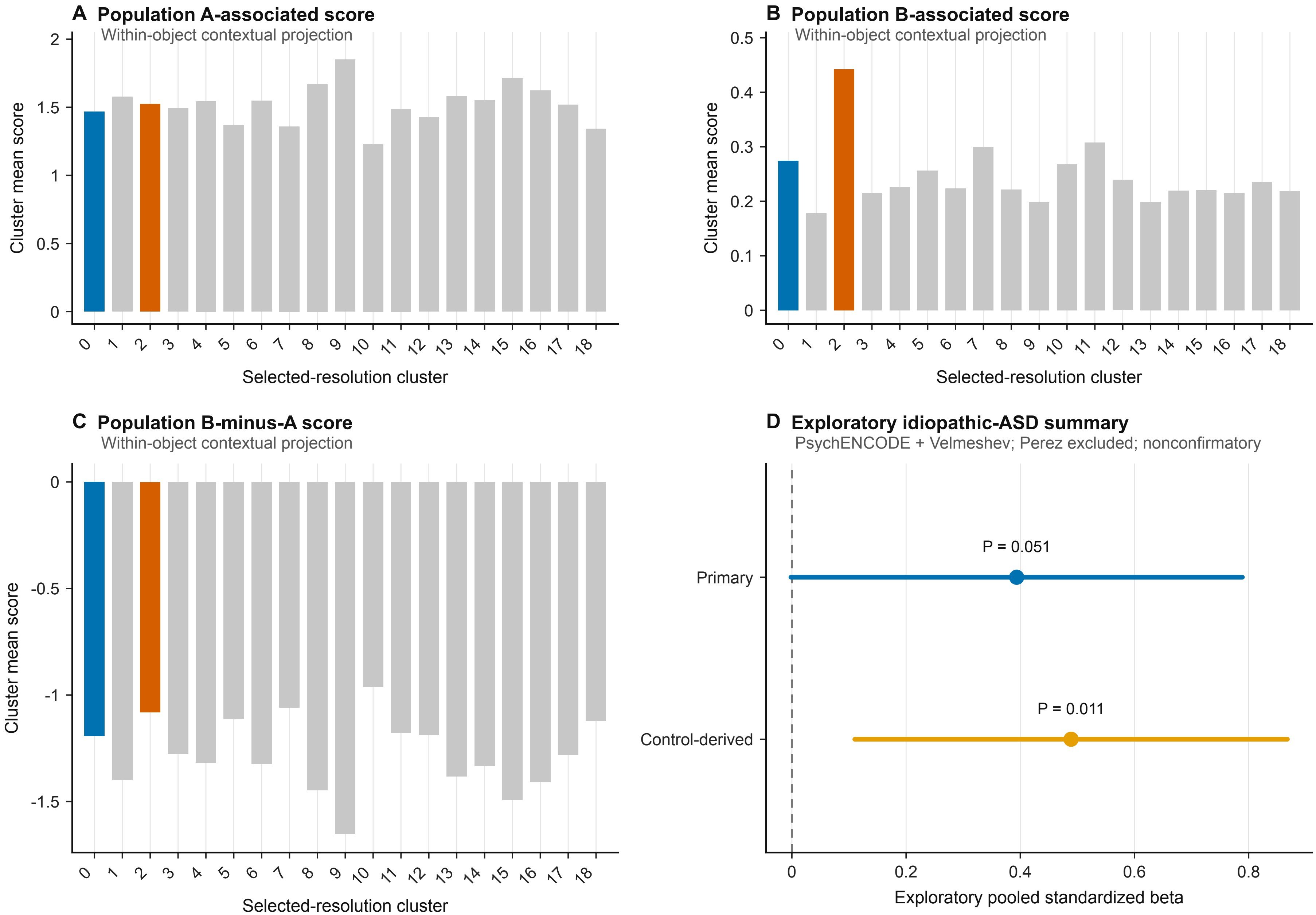

Supplementary Figure S8. Contextual analyses. **(A–C)** Within-object contextual projection of Population A-associated, Population B-associated, and Population B-minus-A scores across all 19 selected-resolution clusters. Bars show cluster mean scores. Population B-associated expression is highest in cluster 2, whereas Population A-associated expression is not unique to cluster 0; this analysis is contextual within the selected-resolution object and supports no broader specificity claim. **(D)** Exploratory fixed-effect standardized summary of PsychENCODE and Velmeshev Population B-minus-A estimates for the primary and control-derived signatures. Points and 95% confidence intervals are on the standardized visualization scale; raw-model *P* values remain the inferential basis in the individual cohorts. Perez is excluded. This pooled summary is exploratory/nonconfirmatory and is outside the prespecified three-test primary BH family.
